# Atypical hepatic hemangioma: imaging features of hyalinized hemangioma

**DOI:** 10.1590/S1679-45082018AI4256

**Published:** 2018-06-05

**Authors:** Letícia Maria Araujo Oliveira Nunes, Caroline Duarte de Mello-Amoedo, Fernando Ide Yamauchi, Ronaldo Hueb Baroni

**Affiliations:** 1 Hospital Israelita Albert Einstein, São Paulo, SP, Brazil.

**Figure 1:**
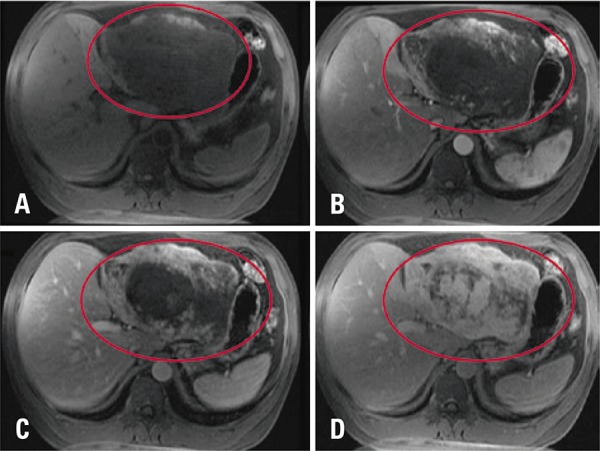
Typical giant hemangioma. Axial sequences of the magnetic resonance in T1-weighted image pre- (A) and post-contrast arterial (B), portal (C) and delayed (D) phases, showing giant hemangioma in segments II and III, with peripheral and discontinued globuliforme-enhancement, and tendency to homogenization

**Figure 2:**
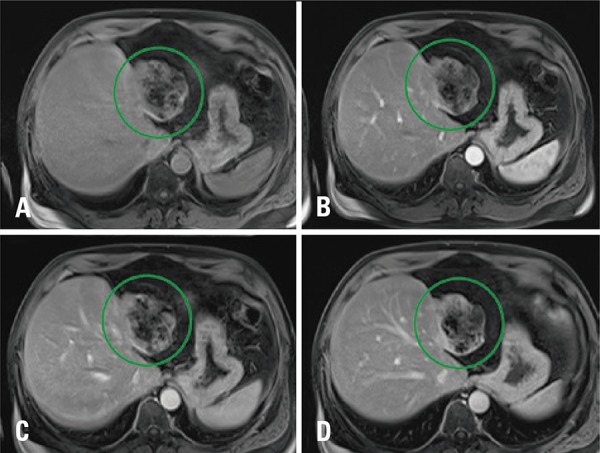
Evolution of hyalinized hemangioma. Axial sequences of the magnetic resonance in T1-weighted image pre- (A) and post-contrast arterial (B), portal (C) and delayed (D) phases, showing volumetric reduction and heterogeneous enhancement pattern

**Figure 3:**
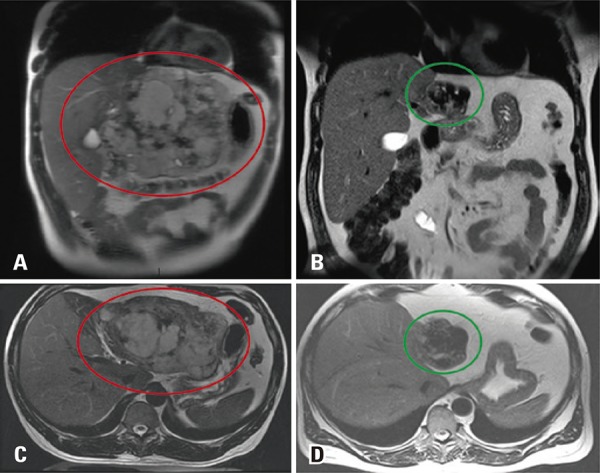
Giant hyalinized hemangioma. Magnetic coronal and axial resonance in T2-weighted image showing characteristic high signal (A and C), and posterior signal and volume reduction after 6 years (B and D) – 17cm to 8cm

A 46 years old man, complaining about abdominal pain, was submmited to a magnetic resonance having a giant liver hemangioma as an incidental finding (Figure 1). In the follow-up, after 6 years, we observed in a T2-weighted sequence volumetric reduction of lesions’ signal in T2-weighted image (Figure 2) was observed. Temporal evolution, associated with imaging features, indicated sclerosed/hyalinized hemangioma (Figure 3).

Hemangioma is the most commonly benign tumor found in the liver with a prevalence between 0.4% to 20%. These lesions have definitive diagnosis by cross-section methods (computed tomography and magnetic resonance imaging), because they present characteristic imaging findings of progressive and discontinued peripheral globuliform enhancement, in addition to high signal on T2-weighted sequences on magnetic resonance imaging. However, atypical liver hemangiomas are difficult to diagnose, and they are caused by complications such as thrombosis, heart failure, hemorrhage, or previous typical sclerosed hemangioma.^(^ ^1^ ^)^

Hyalinized hemangioma can present change in enhancement pattern and characteristic signal, in addition to contour retraction.^(^ ^2^ ^-^ ^5^ ^)^ These cases can be mistaken with other lesions, such as intrahepatic cholangiocarcinoma,^(^ ^6^ ^)^ hepatocellular carcinoma or metastasis.^(^ ^7^ ^)^ Presumptive diagnosis is only possible when previous exams shows temporal evolution of a typical hemangioma.
